# Isolation and identification of *Salmonella* from diarrheagenic infants and young animals, sewage waste and fresh vegetables

**DOI:** 10.14202/vetworld.2015.669-673

**Published:** 2015-05-27

**Authors:** Amruta Nair, T. Balasaravanan, S. V. S Malik, Vysakh Mohan, Manesh Kumar, Jess Vergis, Deepak B. Rawool

**Affiliations:** 1 Division of Veterinary Public Health, Indian Veterinary Research Institute, Bareilly, Uttar Pradesh, India; 2 Department of Biotechnology, Nehru Arts & Science College, Bharathiar University, Coimbatore, Tamil Nadu, India

**Keywords:** *inv* A, isolation, multiplex polymerase chain reaction, *Salmonella*

## Abstract

**Aim:**

This study was carried out to determine the prevalence, distribution, and identification of *Salmonella* serotypes in diarrheagenic infants and young animals, including sewage waste and fresh vegetables.

**Materials and Methods:**

A total of 550 samples were processed for the isolation of *Salmonella* spp., using standard microbiological and biochemical tests. Further polymerase chain reaction (PCR) detection of *Salmonella* genus was carried out using self-designed primers targeting *inv*A gene and thereafter identification of important serotypes namely *Salmonella* Enterica serovar Typhimurium, *Salmonella* Enterica serovar Enteritidis, *Salmonella* Enterica serovar Typhi was performed using published standardized multiplex PCR.

**Results:**

An overall low prevalence of 2.5% (14/550) was observed. The observed prevalence of *Salmonella* spp. in diarrheagenic infants was 1.2% (05/400), diarrheagenic young animals 4% (02/50), sewage waste 10% (05/50), and fresh vegetables 4% (02/50), respectively. In diarrheagenic infants, of the five *Salmonella* isolates identified, two were *Salmonella* Typhimurium, two *Salmonella* Enteritidis, and one was unidentified and hence designated as other *Salmonella* serovar. All the *Salmonella* isolates identified from diarrheagenic young animals and sewage waste belonged to other *Salmonella* serovar, whereas, of the two isolates recovered from fresh vegetables, one was identified as other *Salmonella* serovar, and one as *Salmonella* Typhimurium, respectively.

**Conclusion:**

Isolation of *Salmonella* spp. especially from sewage waste and fresh vegetable is a matter of great concern from public health point of view because these sources can accidentally serve as a potential vehicle for transmission of *Salmonella* spp. to animals and human beings.

## Introduction

Foodborne diseases with its clinical complexities are a potential public health threat worldwide and have a large economic impact across the globe. Foodborne diseases are caused by approximately 250 pathogens including bacteria, viruses, and parasitic organisms [1]. *Salmonella* species are most frequently reported cause of foodborne illness in both humans and animals. The gastroenteritis caused by non-typhoidal *Salmonella* contributes to global public health burden with about 93.8 million cases annually [2]. *Salmonella* infections are largely classified into four clinical types [3]. First, gastroenteritis caused by *Salmonella* Enterica serovar Typhimurium; second, Bacteremia, osteomyelitis, reactive arthritis due to *Salmonella* Typhimurium and *Salmonella* Enteritidis infection; third, enteric fever caused by *Salmonella* Typhi and *Salmonella* Paratyphi and lastly, a carrier state in persons with previous infections [4,5]. Non-typhoidal *Salmonella* is ranked second in its contribution to domestically acquired foodborne illnesses and accounts for 35% of hospitalization and 28% mortality [6]. Of the various *Salmonella* serotypes, *Salmonella* Enteritidis and *Salmonella* Typhimurium are the most common serotypes reported from human clinical cases. In developing countries like India, food-borne illness are mostly under reported; however in the past 29 years (1980-2009) 3485 persons have been affected from 37 *Salmonella* related outbreaks [7].

*Salmonella* infections have been recognized in all the countries, but appear to be more prevalent in areas of intensive animal husbandry, especially poultry, cattle, and pig farming [8]. In general, *Salmonella* contamination is implicated in a wide range of products of animal and plant origin. The primary hosts for non-typhoidal *Salmonella* include cattle, swine, poultry, wild birds (gulls), and pets which excrete this organism in feces, which in turn, can contaminate various food sources and environment [9]. Many asymptomatic livestock play a role as carriers of human pathogens and their feces may contain high concentrations of the organisms which are rarely detected during routine ante-mortem examination [10].

In India, to the best of our knowledge, studies addressing isolation, identification and distribution of *Salmonella* serotypes from varied sources are very few and hence the present study was undertaken with an aim to determine the prevalence and distribution pattern of important *Salmonella* serotypes in diarrheagenic human infants and young animals, sewage waste and fresh vegetables.

## Materials and Methods

### Ethical approval

All the procedures have been carried out in accordance with the guidelines laid down by the Institutional Ethics Committee and in accordance with local laws and regulations. In case of human infants, the diarrheal stool samples were randomly collected, primarily from Medical colleges, pediatric hospitals, and home settings, where the cases of diarrhea were observed. World Health Organization (2009) criteria for acute diarrheal episodes were fulfilled by all the children evaluated at hospitals, health care facilities, and home. Samples were collected only after obtaining informed consent either from the parents of infants or with the help of medical practitioners. The diarrheic fecal samples from young animals were collected from Veterinary dispensaries, organized or unorganized farms after having proper consent from animal owners.

### Collection of samples

A total of 550 samples, from Telangana, Chennai, Maharashtra, Goa, Uttar Pradesh, and Rajasthan comprising of 400 stool samples from diarrheagenic infants (<5 years), 50 fecal samples from diarrheic young animals, 50 samples of fresh vegetables *viz*., mint leaves, tomato, cilantro leaves and 50 samples from sewage, were collected and screened in the present study. All the samples except fresh raw vegetables were collected aseptically using Cary Blair transport swabs (Hi Media Labs, Mumbai, India) and were transported to the laboratory within a week for further isolation and identification studies.

### Isolation of *Salmonella*

Isolation of *Salmonella* was performed as recommended by FDA [11]. In brief, 1ml of the sample from the transport swab was inoculated in 9 ml of buffered peptone water (Hi Media) and incubated at 37°C for 18 h for pre-enrichment. Further, for selective enrichment 0.1 ml of the pre-enriched inoculum was transferred to 10 ml of Rappaport-Vassiliadis broth (Hi Media) and incubated at 42°C for 24 h. After enrichment, a loopful (10 µl) of inoculums was then streaked on xylose lysine desoxycholate (XLD) agar (Hi Media) and incubated at 37°C for 24 h. The presumptive *Salmonella* colonies (4-5 colonies/plate) appearing slightly transparent red halo with a black center surrounded by a pink-red zone on XLD agar were screened further for its biochemical characterization.

### Identification of Salmonella

#### Biochemical characterization

The presumptive colonies of *Salmonella* were further subjected to biochemical tests *viz*., triple sugar iron (TSI), ortho-nitrophenyl galactosidase (ONPG), urease broth, indole, methyl red, Voges-Proskauer and Citrate test (IMViC) as per the standard test protocol described in Bacteriological Analytical Manual FDA [11].

### Genus identification of *Salmonella* isolates by polymerase chain reaction (PCR)

**Figure-1:**
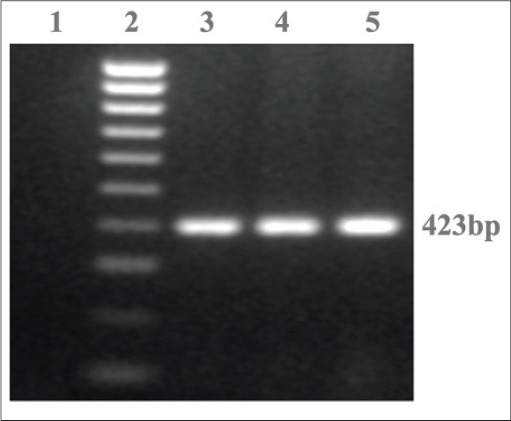
Representative agarose gel analysis of PCR assay targeting invA gene in *Salmonella* isolates. Lane 1: Negative control, Lane 2: 100 bp DNA ladder, Lane 3: *S*. Typhimurium, Lane 4-5: Positive samples

The biochemically positive *Salmonella* isolates were reconfirmed for genus *Salmonella* by employing PCR targeting genus-specific *inv* A gene, with an amplicon size of 423 bp, using a self-designed primers (Table-1). In brief, the DNA was extracted from *Salmonella* isolates using QIAamp DNA extraction kit as per the instructions recommended by the manufacturer. The targeted gene amplification by PCR was carried out with following PCR reaction mixture, which comprised of 2.5 ml of 10× PCR buffer (100 mM Tris-HCl buffer, pH 8.3 containing 500 mM KCl, 15 mM MgCl2, and 0.01% gelatin), 1 ml of 2.5 mM dNTP mix (a final concentration of 1 mM), 1 ml of 50 mM MgCl2, and 10 pmol of forward and reverse primer (Eurofins Pvt. Ltd., Bangaluru), 1 U of Taq DNA polymerase (3B Black Bio, Spain), 4 µl of DNA as a template, and nuclease free water to make up the reaction volume of 25 µL. The PCR amplification was performed in Mastercycler Pro Thermocycler (Eppendorf, Germany). The cycling conditions after gradient PCR were optimized with an initial denaturation at 94°C for 5 min, followed by 35 cycles of denaturation at 94°C for 30 s, annealing at 56°C for 1 min, and extension at 72°C for 1 min 30 s, followed by 10 min of final extension at 72°C and hold at 4°C. The amplified PCR products were resolved by agarose gel electrophoresis, using 1.5% agarose gel stained with ethidium bromide (0.5 mg/ml) and visualized and documented using UV gel documentation system (UVP Gel Seq Software, England). The presence of amplicon at 423 bp was confirmed as *Salmonella* genus (Figure-1).

**Table-1:** Primer details for identification of genus and serotypes of *Salmonella* isolates.

Bacteria	Gene targeted	Primers	Primer sequence	Product size (bp)	Reference
*Salmonella*	*Inv* A	Forward	TCG TGA CTC GCG TAA ATG GCG ATA	423	This study
		Reverse	GCA GGC GCA CGC CAT AAT CAA TAA		
*Salmonella* Enteritidis	*Sdf* I	Forward	TGT GTT TTA TCT GAT GCA AGA GG	304	De Freitas *et al.* 2010
		Reverse	TGA ACT ACG TTC GTT CTT CTG G		
*Salmonella* Typhi	*Via* B	Forward	CAC GCA CCA TCA TTT CAC CG	738	De Freitas *et al.* 2010
		Reverse	AAC AGG CTG TAG CGA TTT AGG		
*Salmonella* Typhimurium	*Spy*	Forward	TTG TTC ACT TTT TAC CCC TGA A	401	De Freitas *et al.* 2010
		Reverse	CCC TGA CAG CCG TTA GAT ATT		

### Serotype identification of *Salmonella* isolates by multiplex PCR

The serotype identification namely *Salmonella* Enteritidis, *Salmonella* Typhi, and *Salmonella* Typhimurium for the confirmed *Salmonella* isolates was carried out using multiplex PCR assay as described earlier [12] with slight modifications. The details of primers, genes targeted and the amplicon size for the serotypes mentioned above are presented in Table-1. In brief, the optimized PCR reaction mixture consisted of 2.5 ml of 10× PCR buffer, 3 µl of 50 mM of MgCl2, 3 µl of 2.5 mM of dNTP mix, 1 U of Taq polymerase, 10 pmol of each set of forward primer, and 10 pmol of each set of reverse primer (Eurofins Pvt. Ltd., Bangaluru), 5 µl of DNA as a template and nuclease free water to make 25 ml of reaction volume. The PCR cycling conditions were programmed with an initial denaturation step of 5 min at 94°C; followed by 35 cycles, each with denaturation at 94°C for 30 s, annealing at 57°C for 1 min., extension at 72°C for 1 min 30 s and finally the final extension was performed at 72°C for 7 min and hold at 4°C. The amplified PCR products were resolved by agarose gel electrophoresis, using 1.5% agarose gel stained with ethidium bromide (0.5 mg/ml) and visualized and documented using UV gel documentation system (UVP Gel Seq Software, England).

## Result

The results of isolation of *Salmonella* spp. and its further serotype identification employing multiplex PCR are presented in Table-2. In brief, on microbiological analysis of 550 samples, 92 samples revealed presumptive *Salmonella* colonies on XLD agar plate. Further, on biochemical characterization, only 14 samples revealed biochemical profile suggestive of *Salmonella* genus. All the 14 isolates were TSI positive, urease negative, ONPG negative, indole negative, methyl red positive, Voges Proskauer negative, and Citrate test positive, respectively. All these biochemically confirmed *Salmonella* isolates were reconfirmed using genus-specific *inv*A gene PCR (Table-2).

**Table-2:** Isolation and identification of *Salmonella* isolates from various sources.

Sampling subjects	Source of sample	Number of sample screened	Positive samples on microbiological analysis	Positive samples on biochemical analysis	Genus specific PCR positive	Serotype identified by multiplex PCR
Human infants (<5 years)	Male	200	23	2	2	*Salmonella* Enteritidis (n=1), other *Salmonella* serotypes (n=1)
	Female	200	30	3	3	*Salmonella* Enteritidis (n=1), *Salmonella* Typhimurium (n=2)
Young animals (<6 months)	Canine	25	8	2	2	Other *Salmonella* serotypes (n=2)
	Bovine	15	3	0	0	ND
	Equine	10	0	0	0	ND
Fresh vegetables	Cilantro leaves	10	5	1	1	*Salmonella* Typhimurium (n=1)
	Tomato	20	3	1	1	Other *Salmonella* serotypes (n=1)
	Mint leaves	20	0	0	0	ND
Sewage waste		50	20	5	5	Other *Salmonella* serotypes (n=5)

ND=Not detected, PCR=Polymerase chain reaction

**Figure-2:**
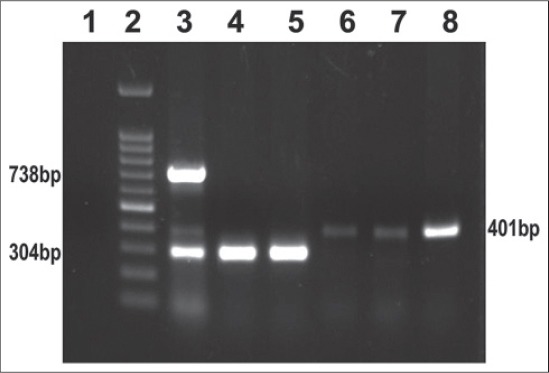
Representative gel analysis of multiplex PCR for identification of *Salmonella* serotypes. Lane 1: Negative control, Lane 2: 100 bp DNA ladder, Lane 3: Standards (*S*. Typhi 738 bp, *S*. Typhimurium 401 bp and *S*. Enteritidis 304 bp), Lane 4 – 5: *S*. Enteritidis isolates, Lane 6 – 8: *S*. Typhimurium isolates

Overall, in the present study a low prevalence of 2.5% (14/550) was observed for *Salmonella* spp. The observed source wise isolation rate of *Salmonella* spp. in diarrheagenic infants was 1.2% (05/400), diarrheagenic young animals 4% (02/50), sewage waste 10% (05/50), and fresh vegetables 4% (02/50), respectively. The details of serotype identified from the respective source are presented in Table-2 and Figure 2.

## Discussion

In developing countries, *Salmonella* is considered to be the prime etiological agent in causing foodborne diseases and childhood morbidity and mortality [2,13]. In the present study, all the screened samples, including infant stools were found negative for *Salmonella* Typhi. This absence of *Salmonella* Typhi in the present study could be due to effective breastfeeding [14,15]. Moreover, it has been reported that the lower incidence of Salmonellosis in Asian countries has been observed in human infants due to their association with higher rates of breastfeeding among their mothers [16,17].

In India, Salmonellosis is endemic in humans, animals, and also associated with foods of animal origin [18-20]. In our study, the overall prevalence of *Salmonella* in diarrheagenic human infants was 1.2% and 4% in diarrheic young animals, which obviously was on the lower side as compared to earlier studies [18-20]. Beside this, in the present study, the serotypes namely *Salmonella* Enteritidis and *Salmonella* Typhimurium were identified from human infants and fresh vegetables. Similar serotype isolation/identification of *Salmonella* Enteritidis and *Salmonella* Typhimurium has also been reported by several authors from human infants and vegetables [21,22]. Almost all the isolates recovered from animals and sewage wastes were considered as other *Salmonella* serotypes because only three important serotypes namely *Salmonella* Enteritidis, *Salmonella* Typhimurium and *Salmonella* Typhi were detected by multiplex PCR employed in the present study. These results are in partial agreement with other authors wherein besides *Salmonella* Enteritidis and *Salmonella* Typhimurium, other *Salmonella* serotypes have also been identified in animals and sewage waste [22,23]. Further, in our study bovine and equine samples failed to yield *Salmonella* isolates, although earlier studies [24,25] have revealed a higher incidence of *Salmonella* spp. in the said species. There can be several factors behind their absence, but the most important would be the limited number of samples screened in the present study and further it has been also reported that diseased animals shed *Salmonella* intermittently, and therefore a minimal 5 consecutive negative fecal cultures is recommended before declaring the animal negative for Salmonellosis [26-28].

In our study, *Salmonella* isolates were also recovered from fresh vegetables (Tomato, Cilantro leaves), which in most of the countries including India are often consumed raw. Thus, the presence of this pathogen in such vegetables is a matter of concern from food safety point of view. Similar reports on detection of *Salmonella* from fresh vegetable have also been reported by several authors [29-32]. In general, these pathogenic bacteria are brought into aquatic environments mainly through treated or untreated wastewater release, surface runoffs, and soil leaching which in turn poses a substantial risk of widespread occurrence of diseases [33]. Besides this, *Salmonella* also have the ability to attach to plant tissue and can survive under adverse temperature conditions due to their effective biofilm formation capability [31,34].

## Conclusion

Isolation of *Salmonella* spp. especially from sewage waste and fresh vegetables is a matter of great concern from public health point of view because these sources can accidentally serve as a vehicle for transmission of *Salmonella* spp. to animals and human beings. However, in the light of results from the present study, epidemiological studies addressing the source of transmission in human and animals needs to devised and executed. Furthermore, more focused intervention studies are required to control this pathogen in sewage waste and fresh vegetables.

## Authors’ Contributions

DBR and TB have designed and supervised the study. AN has carried out bacterial isolation and molecular characterization. TB, VM, MK, and JV have collected the samples and also helped in characterizing the isolates. AN and VM drafted and reviewed the manuscript. DBR and SVSM have edited the manuscript. All authors read and approved the final manuscript.
